# Dysthyroid Optic Neuropathy Dramatically Improved by Sheath-Guided Triamcinolone Orbital Injection: A Case Report

**DOI:** 10.7759/cureus.70437

**Published:** 2024-09-29

**Authors:** Yuji Yamana, Tomoyuki Kashima

**Affiliations:** 1 Oculoplastic Surgery, Oculofacial Clinic Group, Tokyo, JPN

**Keywords:** dysthyroid optic neuropathy, extraocular muscles, triamcinolone, orbital steroid injection, thyroid eye disease

## Abstract

Thyroid eye disease is an autoimmune disorder caused by thyroid-stimulating hormone receptor autoantibodies stimulating the thyroid-stimulating hormone receptor, resulting in proptosis, extraocular muscle dysfunction, diplopia, retro-orbital pain, optic nerve compression, and visual impairment. Dysthyroid optic neuropathy (DON), believed to result from direct compression by enlarged extraocular muscles, represents a severe complication with the potential for irreversible vision loss. Currently, the treatment options for DON are limited to highly invasive procedures, such as orbital decompression surgery and systemic steroid pulse therapy. There are consequently significant challenges in the management of this condition. This report presents a case where a significant improvement in DON was achieved solely through the novel technique of sheath-guided orbital triamcinolone injection, which was utilized with the aim of reaching the deeper part of the orbit under sheath guidance. In this case, the best corrected visual acuity improved from 20/32 (decimal visual acuity: 0.7) to 20/20 (1.2) in the right eye and from 20/500 (0.04) to 20/20 (1.2) in the left eye. Critical flicker frequency values also improved, from 20.3 Hz to 35.0 Hz in the right eye and from 9.3 Hz to 31.5 Hz in the left eye. The cross-sectional areas of the extraocular muscles decreased by an average of 55.5%.

## Introduction

Thyroid eye disease (TED) is a prevalent type of orbital tissue inflammation associated with autoimmune-mediated disorders linked to thyroid dysfunction, most commonly Graves’ disease. These disorders are characterized by a spectrum of metabolic and immunological disturbances [1-5]. TED is a serious ocular condition that can threaten vision. Dysthyroid optic neuropathy (DON), in particular, plays a critical role in causing blindness through the direct compression and stretching of the optic nerve by enlarged extraocular muscles [6,7].

DON is a relatively rare complication, affecting approximately 5% of individuals with Graves’ orbitopathy. According to the 2016 European Thyroid Association/European Group on Graves’ Orbitopathy Guidelines for the Management of Graves’ Orbitopathy (EUGOGO), the recommended first-line treatment involves high-dose intravenous corticosteroids administered over a period of two weeks [1]. In cases where treatment response is inadequate, timely orbital decompression becomes necessary. A small randomized controlled trial indicated that immediate decompression did not yield superior outcomes in patients with DON than first-line treatment with intravenous glucocorticoids [8-12].

While the periocular administration of glucocorticoids is presented as a therapeutic option for TED in the EUGOGO guidelines, it is not indicated for treating DON [1]. However, we have developed a novel injection method to selectively target the extraocular muscles and optic nerve with steroid-sheath-guided orbital triamcinolone injection (SG-OTI) and have attempted to apply this approach when treating DON.

The use of local glucocorticoid administration has been explored since Gebertt first reported this approach in 1961 [13,14]. Localized delivery is typically accomplished through subconjunctival and sub-Tenon injection; however, retro-orbital-septal injection, first introduced in Ebner's report in 2004, offers an alternative method. This procedure is performed transcutaneously using a 12.7-mm, 27-gauge needle, and facilitates the precise delivery of medication into the lateral lower deep orbital fat, yielding promising results in the anti-inflammatory treatment of orbital tissue [15].

Bagheri et al. reported the use of retro-orbital-septal injection of triamcinolone and dexamethasone into both the upper and lower quadrants of the orbital soft tissues [16]. This approach was utilized for patients with active TED who exhibited resistance to or dependence on systemic steroids or who experienced complications arising from systemic steroid use. The results demonstrated a significant reduction in orbital inflammation, including decreased upper and lower eyelid retraction, reduced ocular motility issues (which means avoiding irreversible vision and visual field impairment), and a reduction in inflammatory markers, such as the clinical activity score and NOSPECS classification, which are used to assess the activity of TED. An intriguing aspect of the retro-orbital-septal delivery method is its capacity to produce a dramatic effect after only one or two injections [15,16].

Treatment modalities for DON often include high-dose steroid pulse therapy and orbital decompression, with periocular triamcinolone injections having only a limited effect. However, these techniques still have room for improvement, and a method was developed to achieve the deeper penetration of steroids into the orbit. In this report, we present a case in which significant improvement in the visual acuity of a patient with DON was achieved solely through local steroid administration using our novel method, i.e., SG-OTI.

## Case presentation

Injection procedure

**Figure 1:**
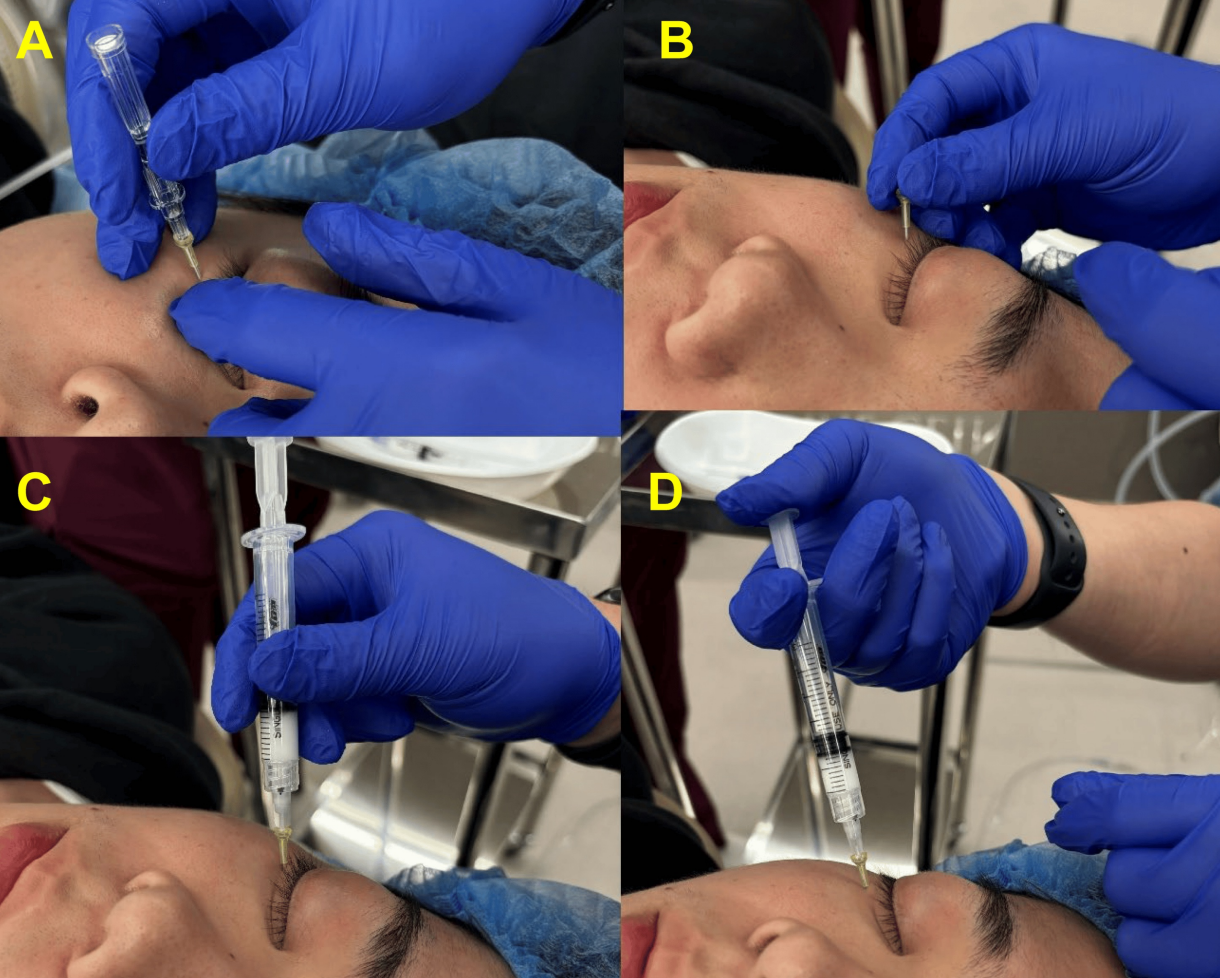
Procedure of sheath-guided orbital triamcinolone injection (SG-OTI). (A) The orbital septum centesis using a 24-gauge 19-mm needle (Dentronics, Tokyo, Japan) with an indwelling cannula. (B) Holding the cannula in position. (C) Passing a 27-gauge 40-mm dull needle (Dentronics) through the cannula to target the orbital muscles. (D) Injecting 1 mL of triamcinolone acetonide (40 mg/mL) further back of the orbital rim.

The SG-OTI procedure entailed first the orbital septum centesis using a 24-gauge 19-mm needle (Dentronics, Tokyo, Japan) with an indwelling cannula, then passing a 27-gauge 40-mm dull needle (Dentronics) through the cannula to target the orbital muscle at four sites (the medial and lateral aspects of the upper and lower eyelids) on top of the application of Emla Cream (a cream containing lidocaine and propitocaine manufactured by Sato Pharmaceutical, Tokyo, Japan). A total of 1 mL of triamcinolone acetonide (40 mg/mL) was subsequently injected inside the orbital rim (Figure 1).

A 66-year-old woman with DON underwent a subtotal thyroidectomy for Plummer's disease (thyroid nodule) one month before presenting to us. Shortly after the surgery, she experienced a deterioration in binocular vision and worsening exophthalmos, leading to a diagnosis of DON by an ophthalmologist and referral to our hospital for treatment. She had a 41-year history of mild thyroid eye disease and a history of sub-total thyroidectomy for hyperthyroidism. Prior to her referral to our hospital, her thyroid eye disease had been stable as mild, and there was no significant treatment history. She had no other comorbidities or any family history of similar conditions.

**Figure 2:**
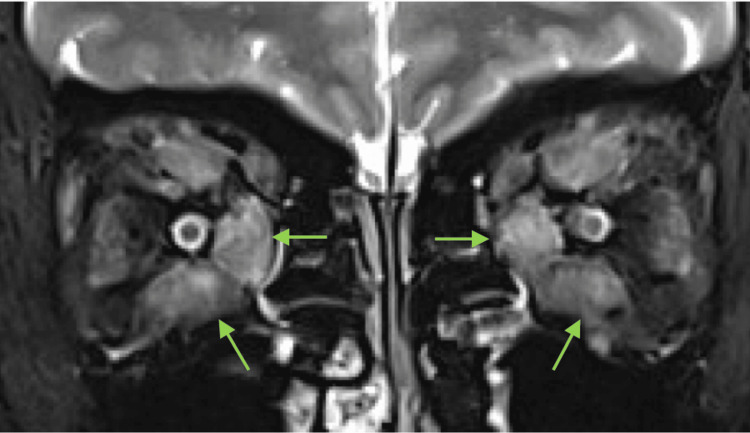
MRI findings of Graves’ orbitopathy with dysthyroid optic neuropathy (T2-weighted and fat-suppressed images using short-tau inversion recovery sequences). Arrows indicate bilateral muscle enlargement, with high signal intensity and more prominent inferior and medial rectus muscles.

At the time of her presentation to our hospital, the patient's subjective symptoms included bilateral blurred vision, difficulty in color discrimination, and retro-ocular pain. The Clinical Activity Score (CAS) score was 7 points. Our initial examination revealed the best corrected visual acuity of 20/32 in the right eye and 20/500 in the left eye. Both optic discs appeared to be swollen, her visual field measured by Goldmann perimetry was constricted in the left eye, and no relative afferent pupillary defect was detected. Hertel measurements were 21 mm in the right eye and 21 mm in the left eye, with limited supraduction in the right eye. The average central critical fusion frequency (CFF) was 20.3 Hz in the right eye and 9.3 Hz in the left. An MRI scan using short-tau inversion recovery sequences revealed bilateral superior, inferior, lateral, medial rectus, and superior oblique muscle enlargement, and high signal intensity (Figure 2).

The patient was injected with 40 mg of triamcinolone using the SG-OTI technique four times, at two-weekly intervals, with follow-up assessments being done two weeks after each injection. Two weeks after the first injection, the patient’s best corrected visual acuity had improved to 20/25 in the right eye and 20/125 in the left eye. Two weeks after the second injection, her visual acuity had improved to 20/20 in the right eye and 20/50 in the left eye. Her average CFF had also improved to 32.6 Hz in the right eye and 23.3 Hz in the left eye. Two weeks after the patient’s third injection, her best corrected visual acuity had improved to 20/20 in the right eye and 20/32 in the left eye. Two weeks after the fourth injection, her best corrected visual acuity had improved to 20/20 in the right eye and 20/20 in the left eye. The patient’s average CFF had again improved to 35.0 Hz in the right eye and 31.5 Hz in the left. In addition, the left eye’s visual field had improved, and the patient’s Hertel measurements were 21 mm in the right eye and 19 mm in the left eye (Table 1). Following the injections, the patient also reported a subjective improvement in her vision, regained the ability to discriminate colors, and experienced relief from retro-ocular pain. No increase in the intraocular pressure was observed during treatment.

**Table 1:** Ophthalmic parameters before and after four sheath-guided orbital triamcinolone injections administered at two-week intervals. BCVA: best corrected visual acuity (decimal visual acuity provided in brackets); CFF: central critical fusion frequency.

	Pre-injection	2 weeks after the first injection	4 weeks after the first injection	6 weeks after the first injection	8 weeks after the first injection
BCVA					
Right	20/32	20/25	20/20	20/20	20/20
Left	20/500	20/125	20/50	20/32	20/20
CFF (Hz)					
Right	20.3			32.6	35.0
Left	9.3			23.3	31.5
Hertel (mm)					
Right	21			20	21
Left	21			20	19

**Figure 3:**
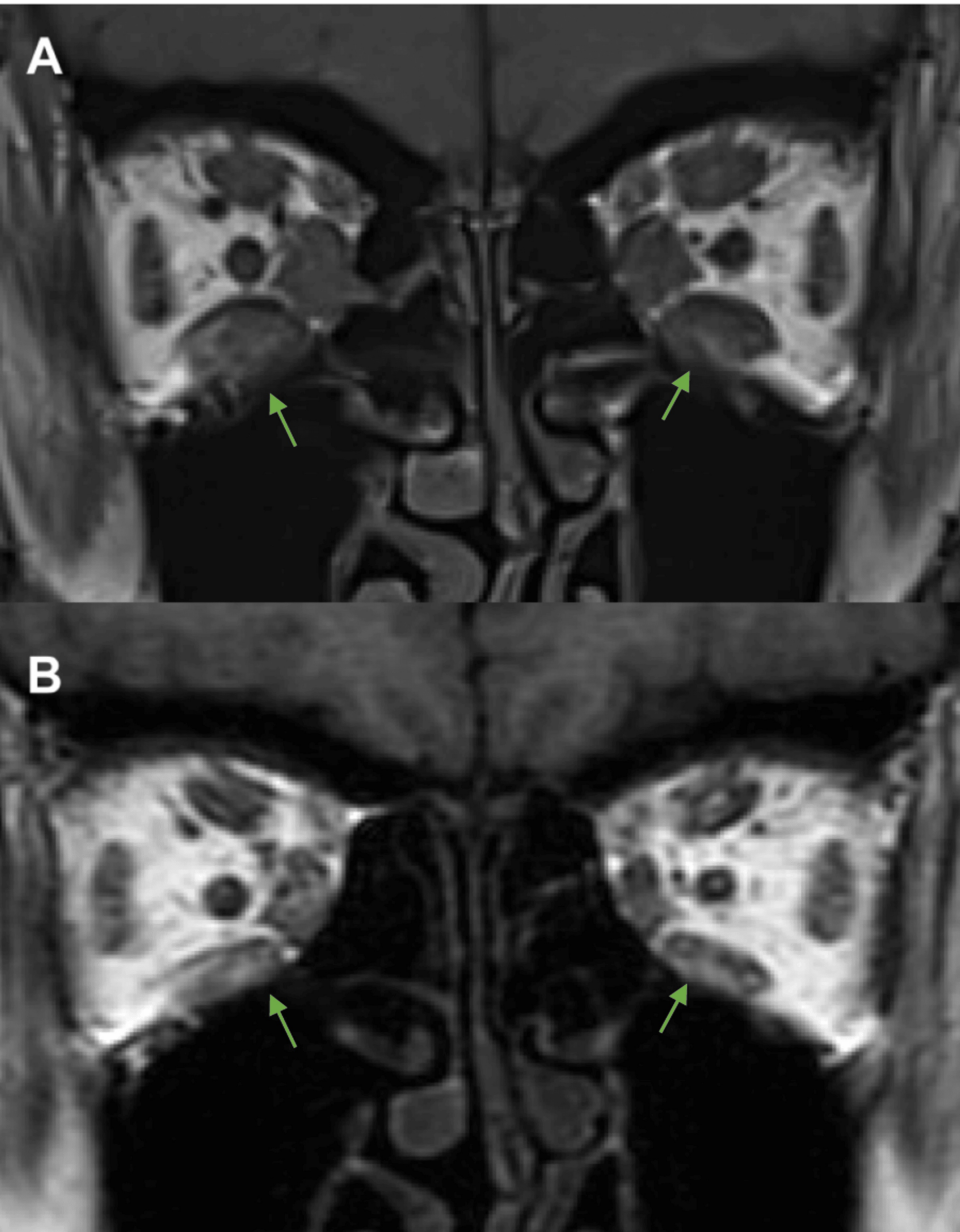
MRI findings (T1-weighted images) of Graves’ orbitopathy with dysthyroid optic neuropathy before (A) and eight weeks after (B) sheath-guided orbital triamcinolone injections. Arrows indicate the decrease in size of the more prominent bilateral inferior rectus muscles.

An MRI scan revealed that the coronal maximum areas of the extraocular muscles eight weeks after the first SG-OTI were smaller than those measured during the patient’s initial pre-injection scan. The area of the superior rectus decreased from 83.6 mm^2^ to 52.8 mm^2^ (percentage of original area: 63.2%) in the right eye and from 93.9 mm^2^ to 49.6 mm^2^ (52.8%) in the left eye. The inferior rectus decreased from 129.9 mm^2^ to 74.05 mm^2^ (57.0%) in the right eye and from 87.9 mm^2^ to 51.6 mm^2^ (58.7%) in the left eye. The medial rectus decreased from 77.9 mm^2^ to 47.2 mm^2^ (60.6%) in the right eye and from 78.4 mm^2^ to 37.3 mm^2^ (47.6%) in the left eye. The lateral rectus decreased from 71.0 mm^2^ to 40.5 mm^2^ (57.0%) in the right eye and from 73.5 mm^2^ to 39.3 mm^2^ (53.5%) in the left eye. The inferior oblique decreased from 29.1 mm^2^ to 15.8 mm^2^ (54.3%) in the right eye and from 26.3 mm^2^ to 13.2 mm^2^ (50.2%) in the left eye (Figure 3).

## Discussion

In cases of optic neuropathy associated with TED, intravenous steroid administration, as recommended by the EUGOGO guidelines, is typically indicated to prevent permanent vision loss. However, complications associated with high-dose steroid administration, contraindications for steroid use, or difficulties in performing surgery due to systemic conditions may necessitate the need for local treatment to control DON.

Local administration of glucocorticoids is generally limited in its effectiveness against DON [1,9-12]. Nevertheless, orbital radiotherapy at a dose of 2000 cGy has demonstrated efficacy in alleviating optic neuropathy comparable to that of high-dose oral steroid treatment (60 mg prednisone per week) [17,18]. Teprotumumab, a monoclonal antibody antagonist to the insulin-like growth factor 1 receptor (IGF-IR), is another alternative therapy, although its application is limited by its high cost [19,20].

In this case of DON, significant reversible improvements in visual acuity and a marked decrease in extraocular muscle cross-sectional areas were achieved solely through local steroid administration using SG-OTI. Previous attempts at local steroid administration may have been ineffective in treating DON because of the difficulty in reaching the deep orbital tissues without piercing the orbital septum, which is necessary for effective steroid pharmacological action on the extraocular muscles and optic nerve. A technique called deep orbital injection, which perforates the orbital septum, has been reported to overcome this drawback [15,16,21-23].

We developed SG-OTI, which involves piercing the orbital septum, to reach deeper into the orbit. Using this new method, we were able to selectively deliver steroids to the enlarged extraocular muscles at the orbital apex, which are implicated in compressing the optic nerve in DON pathology. We believe that this selective action contributed to the observed effects.

The limitations of this report include the short treatment period, the single patient treated, the lack of evaluation regarding the appropriateness of the bi-weekly treatment intervals, and the variability in treatment outcomes due to the SG-OTI technique. It is consequently challenging to generalize the usefulness of SG-OTI for DON based solely on this report. However, further research, including large case series and controlled studies, is necessary to verify the efficacy and safety of SG-OTI. In addition, potential risks associated with SG-OTI, such as damage to the extraocular muscles, optic nerve, and blood vessels, should be investigated.

The potential treatment of DON using SG-OTI expands the therapeutic options available for patients with specific backgrounds. This includes those for whom orbital decompression is contraindicated because of a deterioration in general health, those with immunodeficiencies who must avoid systemic steroid administration, and those who are either young or genetically predisposed to high radiation sensitivity, thus necessitating the avoidance of radiation therapy. TED is a potentially sight-threatening ocular condition, with DON being particularly implicated in causing blindness. Adopting the SG-OTI technique may offer a lower-cost alternative and expand the therapeutic options available for the management of DON.

## Conclusions

In summary, this case report demonstrates favorable treatment outcomes after the use of deep orbital corticosteroid injection as the sole therapy to manage DON. The reversible improvements in visual acuity and reductions in extraocular muscle enlargements achieved without the intervention of other therapies suggest that this is a novel addition to the therapeutic armamentarium for DON. In addition, the treatment outcomes for DON when using SG-OTI suggest that it may offer a viable alternative for patients who are contraindicated for orbital decompression surgery under general anesthesia, immunosuppressive therapy, or radiation therapy. While the short duration of treatment and the limited number of cases present inherent limitations, this case study underscores the necessity for future expansion of case numbers to validate longer-term treatment outcomes.
